# Anticholinergic burden, oral hygiene practices, and oral hygiene status—cross-sectional findings from the Northern Finland Birth Cohort 1966

**DOI:** 10.1007/s00784-020-03485-0

**Published:** 2020-08-03

**Authors:** Antti Tiisanoja, Anna-Maija Syrjälä, Vuokko Anttonen, Pekka Ylöstalo

**Affiliations:** 1 Periodontology and Geriatric Dentistry, Research Unit of Oral Health Sciences, University of Oulu, P.O BOX 5000, FI-90014 Oulu, Finland; 2 Medical Research Center Oulu, Oulu University Hospital and University of Oulu, Oulu, Finland; 3 Cariology, Endodontology and Paediatric Dentistry, Research Unit of Oral Health Sciences, University of Oulu, P.O BOX 5000, FI-90014 Oulu, Finland

**Keywords:** Adults, Anticholinergic drugs, Medication, Dental plaque, Toothbrushing, Oral hygiene

## Abstract

**Objectives:**

To study the association between anticholinergic burden and oral hygiene practices and oral hygiene status among 46-year-old people.

**Materials and methods:**

The study included 1945 participants from the Northern Finland Birth Cohort 1966 (NFBC1966), who had a complete dental status. The participants underwent clinical medical and dental examinations, and their medication data were gathered by combining self-reported drug use with information from the National Prescription Register. Anticholinergic burden was measured using nine previously published anticholinergic scales. Oral hygiene practices were assessed with toothbrushing frequency and oral hygiene status with the presence of visible dental plaque. Poisson regression with robust variance estimation and negative binomial regression models were used to estimate relative risks (RR).

**Results:**

Thirty percent of the participants reported brushing their teeth twice a day and about 25% of their teeth had dental plaque on them. Fifteen percent of the participants used at least one anticholinergic drug or had an anticholinergic burden according to the nine anticholinergic scales*.* After adjustments for confounding factors, the RRs of anticholinergic burden varied between 0.95 and 1.11 for toothbrushing frequency. Anticholinergic burden (according to Anticholinergic Activity Scale, Anticholinergic Cognitive Burden, Chew’s scale) was associated statistically significantly with the number of teeth with dental plaque. For the three scales, RRs varied from 1.24 to 1.50.

**Conclusions:**

Anticholinergic burden associated with poor oral hygiene.

**Clinical relevance:**

The findings stress the importance of providing oral hygiene instructions and prophylactic measures to patients taking anticholinergic drugs.

**Electronic supplementary material:**

The online version of this article (10.1007/s00784-020-03485-0) contains supplementary material, which is available to authorized users.

## Introduction

Good oral hygiene is a major preventive measure against common oral diseases, such as dental caries and periodontal diseases, and it is achieved by brushing teeth twice a day with toothpaste and cleaning the interdental region once a day [1]. Despite this relatively easily achievable goal, many people do not manage to implement sufficient oral hygiene in their daily lives.

Previous studies have shown that multiple sociodemographic and health-related factors, such as income and education level [2], gender [3], unhealthy lifestyle [4], and mental disorders [5, 6] are associated with the quality of oral hygiene. Interestingly, little is known about the effects of medication on oral hygiene, although it has been speculated that drugs could have an impact on oral hygiene through detrimental effects on cognitive or physical functioning [7], or by reducing the rinsing effect of saliva [8].

One medication group that could affect oral hygiene is drugs with anticholinergic properties. This group includes several different drug classes with variation in their indications and anticholinergic potency, for example oxybutynin (urinary incontinence), quetiapine (antipsychotic), and citalopram (antidepressant) [9]. Cumulative exposure to these drugs and their overall anticholinergic effect can be measured with anticholinergic burden scales [9], which identify and score drugs with anticholinergic properties by combining expert opinions, literature reviews, and laboratory measures [9].

Anticholinergic burden has previously been associated with impaired cognitive [10] and physical functioning [9], and with hyposalivation [11]. Based on these findings, it is reasonable to assume that anticholinergic drugs affect oral hygiene. Thus, this paper aimed to study whether anticholinergic burden is associated with oral hygiene practices or oral hygiene status with a hypothesis that individuals with anticholinergic burden have poorer oral hygiene than those without anticholinergic burden.

## Materials and methods

### Participants

The Northern Finland Birth Cohort 1966 (NFBC1966) originally included all children who were from the two northern provinces of Finland and whose expected date of birth was in the year 1966 (*n* = 12,231) [12]. In 2012–2013, individuals living at a convenient distance from the city of Oulu (max. 100 km) were invited to participate in a voluntary 46-year follow-up study (*n* = 3150). About 62% of the invited individuals took part in a clinical oral and medical follow-up examination (*n* = 1962). The final study population included the participants with a complete dental status (*n* = 1945). A written consent for the study was given by all the participants and the study protocol was approved by the Ethical Committee of the Northern Ostrobothnia Hospital District (2/2012). The current study followed the STROBE (Strengthening the Reporting of Observational Studies in Epidemiology) guidelines.

### Lifestyle and general health data

Prior to taking part in the clinical examinations, the cohort members received a questionnaire via post, which included questions about lifestyle, general health, and health behaviours. Questions covered topics such as diagnosed diseases and smoking history, and eating habits. Information from the questionnaire was further complemented with data from the official registers of Oulu University Hospital and the National Institute for Health and Welfare.

### Medication and anticholinergic burden

Each participant’s self-reported prescription drug use was verified with information obtained from the National Prescription Register of the Social Insurance Institute of Finland and the drugs were identified using the Anatomical Therapeutic Chemical (ATC) classification system [13].

Anticholinergic burden from regularly used drugs was measured for each participant using nine different anticholinergic rating scales: Anticholinergic Activity Scale (AAS) [14], Anticholinergic Burden Classification (ABC) [15], Anticholinergic Cognitive Burden (ACB) [16], Anticholinergic Drug Scale (ADS) [17], Anticholinergic Load Scale (ALS) [18], Anticholinergic Risk Scale (ARS) [19], Chew’s scale [20], Clinician-Rated Anticholinergic Scale (CrAS) [21], and Durán’s scale (Durán) [22]. An overview of the scales is presented in Table 1 and a list of all identified anticholinergic drugs is given in Online Resource 1.

**Table 1:** Overview of nine anticholinergic rating scales

Scale	Basis for identification/scoring of anticholinergic drugs	Number of anticholinergic drugs included	Scoring	Grading
Anticholinergic Activity Scale (AAS)	A, B, C	99	0–4	M: 1–3, H: ≥ 4
Anticholinergic Burden Classification (ABC)	A, C	27	0–3	M: 1–2, H: ≥ 3
Anticholinergic Cognitive Burden Scale (ACB)	A, B	88	0–3	M: 1–2, H: ≥ 3
Anticholinergic Drug Scale (ADS)	A, B, C	117	0–3	M: 1–2, H: ≥ 3
Anticholinergic Load Scale (ALS)	A, B	49	0–3	M: 1–2, H: ≥ 3
Anticholinergic Risk Scale (ARS)	A, B	49	0–3	M: 1–2, H: ≥ 3
Chew’s scale (Chew)	A, B, C	107	0–4	M: 1–3, H: ≥ 4
Clinician-Rated Anticholinergic Scale (CrAS)	A, B	60	0–3	M: 1–2, H: ≥ 3
Durán’s scale (Durán)	A, B	100	0–2	M: 1, H: ≥ 2

*M* moderate burden, *H* high burden

*A* expert opinion, *B* literature review/earlier versions of scales, *C* serum anticholinergic activity/radioreceptor assay of anticholinergic activity

All nine anticholinergic scales identify and score drugs with anticholinergic properties by combining expert opinions, literature reviews, and laboratory measures (Table 1). The scales share a similar scoring system, in which the drugs are scored according to their anticholinergic activity and the anticholinergic burden is determined by summing the drugs’ scores. For further information about the anticholinergic scales, the authors recommend a recent systematic review by Villalba-Moreno and colleagues [23]. Anticholinergic burden was used in the analyses both as categorical variable (yes/no) and continuous variable to depict total anticholinergic burden.

### Clinical oral examination

All the oral examinations were carried out following a standardized study protocol by seven trained and calibrated dentists. The examinations consisted of cariological, periodontal, and oral physiological segments that have been presented in detail earlier [24]. The window of time for the examinations was between April 2012 and June 2013 and therefore training and calibration of the dentists’ study were repeated every 3 months. Clinical oral examinations were performed in a dental office with modern dental unit with an oral mirror, WHO ball-pointed gingival probe, and fibre-optic transillumination. During the field phase, each examiner re-examined 10 participants 1 month after the previous examination in order to assess intraexaminer agreement, and a gold-standard dentist performed a parallel examination for about seven participants of each examiner to assess interexaminer agreement. While attending the clinical oral examination, the participants also responded to a computer-aided dental survey.

### Oral hygiene practices and oral hygiene status

Both oral hygiene practices and oral hygiene status were used as outcomes in the current study. Oral hygiene practices were assessed with toothbrushing frequency and oral hygiene status with the number of teeth with visible dental plaque.

Toothbrushing and its frequency was determined with the dental survey, which included the question: “Do you brush your teeth” with the options of: “Very rarely/sometimes within a week/every now and then/once a day/twice a day/more than twice a day?” The answers were classified into two categories: toothbrushing at least twice a day vs. toothbrushing less than twice a day.

Dental plaque was assessed during the clinical oral examination. Before the actual examination the teeth were air-dried, but not professionally cleaned. Dental plaque was examined visually and with a probe from the buccal surface of each tooth, excluding 3rd molars. If visible dental plaque was detected on the tooth’s buccal surface, it was included in the number of teeth with dental plaque. In addition to continuous variable, dental plaque was also used as categorical variable:

$$ \left(\frac{\mathrm{number}\ \mathrm{of}\ \mathrm{teeth}\ \mathrm{with}\ \mathrm{dental}\ \mathrm{plaque}}{\mathrm{number}\ \mathrm{of}\ \mathrm{teeth}\ }\right)\ast 100 $$

This variable was classified into two categories according to the distribution curve (the highest quarter): plenty of dental plaque (≥ 36% of teeth covered with dental plaque) vs. less dental plaque (< 36% of teeth covered with dental plaque).

### Potential confounding factors

Sociodemographic factors were asked in the postal questionnaire. For the analyses, participant’s education level was categorized into two categories: having a higher education (university level or equivalent) vs. having a lower education. Marital status was also categorized into two categories: being married/in an equivalent relationship vs. not married. Smoking was also asked in the postal questionnaire and it was categorized as non-smokers (never smokers and those who had quit) vs. current smokers.

Alcohol consumption was assessed according to participants’ answers on questions related to alcohol drinking. The questions covered both consumption frequency and single time amounts of mild, moderate, and strong alcohol beverages. The answers were converted into pure alcohol consumption per day (g/day) and the WHO-recommended risk level for moderate alcohol consumption was used as the cut-off value (women ≥ 20 g/day and men ≥ 40 g/day) [25].

Information for mental health disorders (depression and psychosis) was gathered from medication data, the national prescription register, medical registers, and a 12-item General Health Questionnaire (GHQ-12) [26]. Participant’s general diseases were verified from the postal questionnaire, the medical records, and official prescription registers. Six general diseases or disease groups were taken into account: congestive heart failure, coronary heart disease, diabetes (type I and II), rheumatic diseases (rheumatoid arthritis, Sjögren’s syndrome, Polymyalgia rheumatica), epilepsy, and stroke. A number of general diseases were used in the analyses.

### Statistical methods

Toothbrushing, as a categorical variable, and the number of teeth with dental plaque, as both continuous and categorical variable, were used as outcome variables. Due to the small number of participants with high anticholinergic burden, moderate and high anticholinergic burdens were combined into one category.

Relative risks (RR) with 95% confidence intervals (CI) were estimated using a Poisson regression model with a robust variance estimation (categorical variables) and using a negative binominal regression model (continuous variables). The selection of potential confounders was based on the literature and all the analyses were adjusted for gender, marital status, education, alcohol consumption, smoking, use of removable prostheses, depression, psychosis, and general diseases. An offset variable (number of teeth) was used in the analyses for dental plaque. SPSS software (version 24.0, Chicago, IL, USA) was used in all the statistical analyses.

## Results

All the participants (*n* = 1945) were 45–47 years old at the time of the follow-up study and 54% of them were women (*n* = 1041). Most of the participants were married or in equivalent relationship (76%) and 27 % of the participants had a higher education (university or equivalent). Characteristics of the whole study population are presented in Table 2*.* The participants had on average 27 teeth (± SD 2.1, 3rd molars excluded) and about 25% of the teeth had dental plaque on them (the median number of teeth with dental plaque was about seven) (Table 2). Thirty percent of the participants (*n* = 586) reported that they brush their teeth less than twice a day.

**Table 2:** Characteristics of the study population

Variable	All participants	Anticholinergic burden
*n*	1945	282
Gender, proportion of women, *n* (%)	1041 (54)	183 (65)
Married or equivalent relationship, *n* (%)	1482 (76)^a^	195 (69)^a^
Higher education (university or equivalent), *n* (%)	533 (27)^b^	60 (21)^b^
Current smoker, *n* (%)	424 (22)	79 (28)
Moderate alcohol consumption risk, *n* (%)	182 (9.4)^c^	35 (12)^c^
Diagnosed depression or depressive symptoms, *n* (%)	145 (7.5)	66 (23)
Diagnosed psychosis, *n* (%)	30 (1.5)^d^	23 (8.0)^d^
One or more general diseases, *n* (%)	174 (9.0)	87 (31)
Epilepsy, *n* (%)	26 (1.3)	16 (5.7)
Coronary heart disease	19 (1.0)	7 (2.5)
Congestive heart failure	8 (0.4)	6 (2.1)
Stroke	18 (0.9)	9 (3.2)
Rheumatic disease	52 (2.7)	26 (9.2)
Diabetes	67 (3.4)	37 (13)
Total number or drugs, median (IQR)	0 (0–1)	2 (1–3)
Participants using at least one regular drug, *n* (%)	731 (38)	282 (100)
Using at least one anticholinergic drug, *n* (%)	282 (15)	282 (100)
Anticholinergic Activity Scale, *n* (%)		
≥ 1	106 (5.4)	106 (38)
Anticholinergic Burden Classification, *n* (%)		
≥ 1	36 (1.8)	36 (13)
Anticholinergic Cognitive Burden Scale, *n* (%)		
≥ 1	123 (6.3)	123 (44)
Anticholinergic Drug Scale, *n* (%)		
≥ 1	153 (7.9)	153 (54)
Anticholinergic Load Scale, *n* (%)		
≥ 1	180 (9.3)	180 (64)
Anticholinergic Risk Scale, *n* (%)		
≥ 1	73 (3.7)	73 (26)
Chew’s scale, *n* (%)		
≥ 1	168 (8.7)	168 (60)
Clinician-Rated Anticholinergic Scale, *n* (%)		
≥ 1	118 (6.0)	118 (42)
Durán’s scale, *n* (%)		
≥ 1	139 (7.1)	139 (49)
Number of teeth (excluding 3rd molars), mean (± SD)	27 (2.1)	26 (2.0)
Using a removable prosthesis, *n* (%)	19 (1.0)	6 (2)
Number of teeth with dental plaque, median (IQR)	6.6 (1–10)^e^	7.5 (1–11)^e^
Percentage of teeth covered with dental plaque, on average	24	29
Toothbrushing less than twice a day, *n* (%)	586 (30)^a^	84 (30)^a^

*SD* standard deviation, *IQR* inter quartile range

^a^56/11 missing

^b^80/14 missing

^c^55/9 missing

^d^64/9 missing

^e^11/4 missing

Seven hundred thirty-one participants (38%) used at least one regular drug and 282 participants (~ 15%) used at least one anticholinergic drug included in the nine anticholinergic scales or had an anticholinergic burden. About 5% of the participants had moderate anticholinergic burden and only 1.5% of the participants had a high anticholinergic burden according to the scales.

The results of the univariate analyses are presented in Table 3 and the results of the multivariate regression analyses in Table 4. After adjustments for confounding factors, RRs for infrequent toothbrushing varied from 0.95 (ACB) to 1.11 (Durán) (Table 4). However, none of the RRs was statistically significant at the *p* value level 0.05 (Table 4). With continuous anticholinergic burden, the RRs varied from 0.96 (ARS) to 1.08 (Durán).

**Table 3:** Unadjusted associations between independent variables and oral hygiene

Variables	Number of teeth with dental plaque (RR, CI 95%)^a^	Toothbrushing less than twice a day (RR, CI 95%)^a^	Plenty of dental plaque (RR, CI 95%)^a^
Gender			
Women	1.0	1.0	1.0
Men	1.41 (1.29–1.55)	2.51 (2.15–2.92)	1.76 (1.05–2.06)
Married or equivalent relationship			
Yes	1.0	1.0	1.0
No	1.09 (0.98–1.23)	1.29 (1.11–1.51)	1.05 (0.87–1.26)
Higher education (university or equivalent)			
Yes	1.0	1.0	1.0
No	1.15 (1.02–1.30)	1.97 (1.61–2.39)	1.28 (1.06–1.55)
Current smoker			
No	1.0	1.0	1.0
Yes	1.22 (1.10–1.36)	1.38 (1.19–1.60)	1.45 (1.24–1.71)
Moderate alcohol consumption risk^b^			
No	1.0	1.0	1.0
Yes	1.22 (1.04–1.42)	1.13 (0.91–1.41)	1.46 (1.18–1.81)
Number of general diseases (continuous)	1.23 (1.04–1.44)	1.26 (1.07–1.49)	1.31 (1.16–1.48)
Diagnosed depression or depressive symptoms			
No	1.0	1.0	1.0
Yes	1.08 (0.92–1.28)	0.99 (0.76–1.30)	1.12 (0.86–1.47)
Diagnosed psychosis			
No	1.0	1.0	1.0
Yes	1.91 (1.45–2.52)	1.53 (1.02–2.29)	2.35 (1.72–3.23)
Using removable prothesis			
No	1.0	1.0	1.0
Yes	1.99 (1.45–2.72)	1.99 (1.37–2.90)	2.87 (2.13–3.86)
Anticholinergic Activity Scale			
0	1.0	1.0	1.0
≥ 1	1.58 (1.24–2.02)	1.13 (0.84–1.52)	1.52 (1.17–1.97)
Continuous	1.14 (1.06–1.21)	1.04 (0.94–1.14)	1.13 (1.04–1.22)
Anticholinergic Burden Classification			
0	1.0	1.0	1.0
≥ 1	1.29 (0.85–1.93)	1.19 (0.75–1.88)	1.74 (1.03–2.94)
Continuous	1.09 (0.99–1.20)	1.04 (0.88–1.23)	1.15 (0.99–1.33)
Anticholinergic Cognitive Burden			
0	1.0	1.0	1.0
≥ 1	1.37 (1.09–1.71)	1.11 (0.85–1.46)	1.38 (1.06–1.79)
Continuous	1.16 (1.09–1.24)	1.05 (0.95–1.16)	1.17 (1.08–1.27)
Anticholinergic Drug Scale			
0	1.0	1.0	1.0
≥ 1	1.32 (1.07–1.62)	1.14 (0.89–1.46)	1.29 (1.00–1.64)
Continuous	1.13 (1.05–1.21)	1.06 (0.96–1.16)	1.13 (1.03–1.23)
Anticholinergic Load Scale			
0	1.0	1.0	1.0
≥ 1	1.25 (1.03–1.51)	1.13 (0.90–1.42)	1.26 (1.00–1.59)
Continuous	1.08 (0.99–1.18)	1.04 (0.93–1.17)	1.07 (0.95–1.21)
Anticholinergic Risk Scale			
0	1.0	1.0	1.0
≥ 1	1.45 (1.09–1.92)	1.16 (0.83–1.62)	1.39 (1.01–1.93)
Continuous	1.12 (1.09–1.23)	1.02 (0.88–1.17)	1.09 (0.96–1.24)
Chew’s scale			
0	1.0	1.0	1.0
≥ 1	1.51 (1.24–1.84)	1.22 (0.97–1.53)	1.46 (1.17–1.82)
Continuous	1.11 (1.04–1.18)	1.05 (0.97–1.14)	1.09 (1.01–1.18)
Clinician-Rated Anticholinergic Scale			
0	1.0	1.0	1.0
≥ 1	1.32 (1.04–1.66)	1.15 (0.88–1.50)	1.33 (1.01–1.74)
Continuous	1.13 (1.04–1.23)	1.02 (0.90–1.14)	1.12 (1.01–1.25)
Durán’s scale			
0	1.0	1.0	1.0
≥ 1	1.27 (1.03–1.58)	1.16 (0.90–1.50)	1.20 (0.92–1.57)
Continuous	1.13 (1.03–1.25)	1.10 (0.98–1.24)	1.11 (0.97–1.27)

^a^Data presented as a relative risk (RR) with 95% confidence intervals (CI 95%)

^b^Moderate risk for alcohol consumption: ≥ 20 g of pure ethanol/day (women), ≥ 40 g of pure ethanol/day (men)

**Table 4:** Adjusted associations between anticholinergic scales and oral hygiene

Anticholinergic Scale	Number of teeth with dental plaque (RR, CI 95%)^a^	Toothbrushing less than twice a day (RR, CI 95%)^a^	Plenty of dental plaque (RR, CI 95%)^a^
Anticholinergic Activity Scale			
0	1.0	1.0	1.0
≥ 1	1.50 (1.16–1.94)*	1.09 (0.81–1.48)	1.51 (1.11–2.06)*
Continuous	1.13 (1.02–1.24)*	1.02 (0.92–1.15)	1.09 (0.97–1.21)
Anticholinergic Burden Classification			
0	1.0	1.0	1.0
≥ 1	1.17 (0.78–1.75)	1.04 (0.63–1.72)	1.15 (0.70–1.88)
Continuous	1.08 (0.93–1.24)	1.03 (0.86–1.23)	1.10 (0.94–1.27)
Anticholinergic Cognitive Burden			
0	1.0	1.0	1.0
≥ 1	1.24 (0.97–1.57)	0.95 (0.71–1.26)	1.15 (0.85–1.57)
Continuous	1.12 (1.00–1.24)*	0.98 (0.86–1.10)	1.08 (0.97–1.21)
Anticholinergic Drug Scale			
0	1.0	1.0	1.0
≥ 1	1.22 (0.98–1.52)	1.09 (0.84–1.40)	1.20 (0.90–1.59)
Continuous	1.09 (0.98–1.23)	1.02 (0.91–1.15)	1.04 (0.92–1.18)
Anticholinergic Load Scale			
0	1.0	1.0	1.0
≥ 1	1.13 (0.93–1.39)	1.07 (0.83–1.36)	1.17 (0.90–1.54)
Continuous	1.03 (0.92–1.15)	1.03 (0.91–1.17)	1.00 (0.87–1.15)
Anticholinergic Risk Scale			
0	1.0	1.0	1.0
≥ 1	1.25 (0.92–1.71)	1.00 (0.70–1.43)	1.01 (0.68–1.50)
Continuous	1.03 (0.90–1.18)	0.96 (0.82–1.13)	0.97 (0.84–1.13)
Chew’s scale			
0	1.0	1.0	1.0
≥ 1	1.36 (1.10–1.68)*	1.06 (0.82–1.35)	1.36 (1.04–1.78)*
Continuous	1.08 (0.99–1.18)	1.04 (0.94–1.16)	1.03 (0.93–1.14)
Clinician-Rated Anticholinergic Scale			
0	1.0	1.0	1.0
≥ 1	1.19 (0.93–1.52)	1.07 (0.81–1.42)	1.13 (0.83–1.54)
Continuous	1.07 (0.96–1.20)	0.97 (0.86–1.09)	1.03 (0.92–1.17)
Durán’s scale			
0	1.0	1.0	1.0
≥ 1	1.18 (0.94–1.49)	1.11 (0.84–1.46)	1.02 (0.75–1.40)
Continuous	1.08 (0.93–1.24)	1.08 (0.94–1.25)	0.99 (0.85–1.15)

^a^Data presented as a relative risk (RR) with 95% confidence intervals (CI 95%)

*p < 0.05

All the models were adjusted for gender, marital status, education, alcohol consumption, smoking, the use of removable prostheses, depression, psychosis, and general diseases. In addition, the number of teeth was used as an off-set variable in the analyses for dental plaque

Participants with anticholinergic burden had a higher likelihood of having more teeth with dental plaque than those without the burden (Table 4). RRs for the number of teeth with dental plaque were between 1.13 and 1.50 for categorical explanatory variables; for continuous explanatory variables, RRs were between 1.03 and 1.13. The strongest association was when anticholinergic burden was measured with the AAS and Chew’s scale (RR: 1.50, CI: 1.16–1.96; RR: 1.36, CI: 1.10–1.68, respectively). With continuous anticholinergic burden, AAS and ACB (RR: 1.13, CI: 1.02–1.24, RR: 1.12, CI: 1.00–1.24, respectively) were associated with the number of teeth with dental plaque (Table 4). The results using categorical measure for dental plaque did not differ significantly and are presented in the Table 4.

Additional analyses were also done using a population where participants with psychiatric diseases were excluded. The results of those analyses where participants with psychiatric diseases were excluded did not essentially differ from those obtained in the main analyses.

## Discussion

It appears that anticholinergic burden is associated with poor oral hygiene status, but not with oral hygiene practices.

Anticholinergic drugs could affect oral hygiene in several ways. Firstly, it can be speculated that the anticholinergic burden decreases participant motivation and ability to remember to brush their teeth twice a day due to a decline in cognitive functions [9]. Secondly, anticholinergic burden may lead to insufficient removal of dental plaque by lowering the patient’s attention and precision required to carry out efficient toothbrushing [27]. Thirdly, it could also be speculated that the effects of anticholinergic burden on oral hygiene are mediated by hyposalivation, which is thought to promote the development of dental plaque by diminishing bacterial clearance from the oral cavity, by increasing bacterial adherence to hard and soft tissues, and by reducing anti-bacterial effects of saliva [28]. It should be emphasized that the current evidence on the topic of hyposalivation is inconclusive, as there are studies both for [29–32] and against [33, 34].

A major strength of the study was the large, unselected population with an acceptable participation rate (61.7%), which meant that the study population was a good representation of this age-group living in Northern Finland. On the other hand, it should also be kept in mind that despite the fairly good participation rate, 38% drop-out of participants from the clinical oral examination may have led to sample selection bias.

Another strength of the present study was the use of two outcomes: self-reported toothbrushing and the presence of dental plaque. In this population, there were discrepancies between the self-reported toothbrushing frequency and the presence of dental plaque, potentially indicating a high risk of social desirability bias related to the toothbrushing variable. It is self-evident that the dental plaque variable provides more accurate information about the level of oral hygiene.

The assessment of regular drug use can also be seen as one of the strengths of this study. The use of drugs during the years 2012 and 2013 was determined by combining self-reported drug use and drug purchase information from the National Prescription Register. Despite this comprehensive approach, there are factors, such as non-adherence, that cannot be entirely excluded. However, previous studies have shown that the quantification method used is sufficient for the present study [35, 36].

The disadvantage of this population was that there was a fairly small number of participants with anticholinergic burden. It can be speculated that the associations would be easily detectable in a population with a higher drug usage, but—at the same time—the risk of possible confounding due to co-morbidities would most likely be high.

As suggested by a recent review [37], anticholinergic burden in the present study was measured by using several anticholinergic scales in order to increase comparability with other studies. The rationale for this approach is the considerable variation between individual anticholinergic scales, for example in the inclusion and ranking of anticholinergic drugs [23, 37]. It should be kept in mind that there are also certain limitations in the anticholinergic scales. The scales assume that pharmacological mechanisms are simple and that all the drugs have a linearly additive anticholinergic effect [23]. Furthermore, the scales ignore intrinsic factors such as pharmacokinetics and susceptibility to anticholinergic effects [37]. None of the scales used takes drug dosage into account. Despite the aforementioned, the anticholinergic scales used are so far the best available method to measure anticholinergic burden.

Although multiple potential confounding factors were taken into account, it is still possible that other, uncontrolled factors confound the association between anticholinergic burden and oral hygiene*.* These could be behavioural factors, such as diet, or conditions effecting indirectly oral hygiene via hyposalivation such as the radiotherapy of the head and neck region, for example. It should also be kept in mind that medical conditions as a cause of anticholinergic drug use may also affect oral hygiene. On the other hand, it can be speculated that these effects may not have essential biasing effect due to the fact that major medical conditions were taken into account in the analyses. In complementary analyses, it was observed that the exclusion of participants with psychiatric diseases did not change the results essentially (data not shown).

From a clinical perspective, the findings stress the importance of providing oral hygiene instructions to patients taking anticholinergic drugs. In addition to having a dry mouth, these patients seem to be at a higher risk of having poor oral hygiene. A combination of these conditions can have serious harmful effects on oral health and thus, dental professionals should always give instructions to this patient group on how to achieve sufficient oral hygiene and how to manage with dry mouth and its symptoms. It should also be kept in mind that the need for other prophylactic measures (i.e. additional fluoride) is high among patients with dry mouth.

## Conclusion

Anticholinergic burden is associated with poor oral hygiene.

## Electronic supplementary material

ESM 1 (PDF 110 kb)
