# Supplementary material for: Anticholinergic burden, oral hygiene practices, and oral hygiene status—cross-sectional findings from the Northern Finland Birth Cohort 1966
Source: Clin Oral Investig. 2020 Aug 3;25(4):1829–37. doi: 10.1007/s00784-020-03485-0 (PMC7966223; doi:10.1007/s00784-020-03485-0)
Supplement: Supplementary file 1 — (PDF 110 kb) [file 784_2020_3485_MOESM1_ESM.pdf]

ESM 1 Drugs with anticholinergic properties identified in the study

Drug name	ATC-code	n
Metformin	A10BA02 / A10BD07	25
Citalopram	N06AB04	19
Lansoprazole	A02BC03	15
Cetirizine	R06AE07	11
Fluticasone/salmeterol	R03AK06 / R01AD58	11
Methotrexate	L04AX03	11
Escitalopram	N06AB10	10
Fluoxetine	N06AB03	10
Mirtazapine	N06AX11	10
Codeine	N02AJ06 / N02AA59	9
Metoprolol	C07AB02 / C07BB02	9
Prednisolone	H02AB06	8
Amitriptyline	N06AA09 / N06CA01	7
Venlafaxine	N06AX16	7
Oxazepam	N05BA04	6
Olanzapine	N05AH03	6
Duloxetine	N06AX21	5
Oxcarbazepine	N03AF02	5
Quetiapine	N05AH04	5
Tramadol	N02AX02	5
Paroxetine	N06AB05	4
Sertraline	N06AB06	3
Valproic acid	N03AG01	3
Azathioprine	L04AX01	2
Bupropion	A08AA62 / N06AX12	2
Celecoxib	M01AH01	2
Ciclosporin	L04AD01	2
Doxepin	N06AA12	2
Furosemide	C03CA01	2
Loratadine	R06AX13	2
Sumatriptan	N02CC01	2
Tizanidine	M03BX02	2
Carbamazepine	N03AF01	1
Clonazepam	N03AE01	1
Fexofenadine	R06AX26	1
Fentanyl	N02AB03	1
Haloperidol	N05AD01	1
Levomepromazine	N05AA02	1
Nortriptyline	N06AA10	1
Orphenadrine	M03BC01 / M03BC51	1
Pramipexole	N04BC05	1
Risperidone	N05AX08	1
Solifenacin	G04BD08	1
Topiramate	N03AX11	1
Warfarin	B01AA03	1

Anatomical Therapeutic Chemical (ATC)
